# STtools: a comprehensive software pipeline for ultra-high-resolution spatial transcriptomics data

**DOI:** 10.1093/bioadv/vbac061

**Published:** 2022-09-01

**Authors:** Jingyue Xi, Jun Hee Lee, Hyun Min Kang, Goo Jun

**Affiliations:** Department of Biostatistics, School of Public Health, University of Michigan, Ann Arbor, MI 48109, USA; Department of Molecular & Integrative Physiology, University of Michigan Medical School, Ann Arbor, MI 48109, USA; Department of Biostatistics, School of Public Health, University of Michigan, Ann Arbor, MI 48109, USA; Department of Epidemiology, Human Genetics & Environmental Sciences, School of Public Health, University of Texas Health Science Center at Houston, Houston, TX 77030, USA

## Abstract

**Motivation:**

While there are many software pipelines for analyzing spatial transcriptomics (ST) data, few can process ultra-high-resolution datasets generated by emerging technologies. There is a clear need for new software tools that can handle submicrometer resolution ST data with computational scalability without compromising its resolution.

**Results:**

We developed STtools, a software pipeline that provides a versatile framework to handle ST datasets with various resolutions, such as the ones produced by Seq-Scope (<1 μm), Slide-seq (10 μm) and VISIUM (100 μm). It automatically processes raw FASTQ files and runs downstream analyses at several folds higher resolution than existing methods. It also generates various visualizations including transcriptome density, cell type mapping, marker gene highlighting and subcellular architectures.

**Availability and implementation:**

STtools is publically available for download at https://github.com/seqscope/STtools

**Supplementary information:**

Supplementary data are available at *Bioinformatics Advances* online.

## 1 Introduction

Recent developments in single-cell and spatial RNA-sequencing technologies enabled fine-scale exploration of cell-type-specific expressions and tissue compositions. Technologies such as VISIUM (Ståhl *et al.*, 2016), Slide-seq (Rodriques *et al.*, 2019; Stickels *et al.*, 2021) and Seq-Scope (Cho *et al.*, 2021) associates specific barcode sequences with spatial coordinates and attaches these spatial barcodes to individual cDNA fragments to resolve transcriptomic profiles with spatial resolution.

Current software tools analyzing spatially resolved transcriptomes (10x Genomics, 2022; Palla *et al.*, 2022; Petukhov *et al.*, 2021) are primarily designed for relatively coarse resolution technologies such as VISIUM (100 μm) or Slide-seq (10 μm), where each spatial barcode typically represents more than single cells. However, when analyzing transcriptome spatially resolved at a micrometer or a submicrometer resolution, most current tools perform poorly due to various computational challenges. First, the number of spatial barcodes per mm^2^ rapidly increases as resolution increases (∼120 for VISIUM, ∼3K for Slide-seq and >1M for Seq-Scope), and few tools seamlessly scale to handle millions of spatial barcodes. Second, even though higher-resolution technologies may contain larger Universal Molecular Identifier (UMI) counts per given area, the UMI count per spatial barcode is typically much lower due to the limited number of mRNAs that can be captured. As a result, existing tools may perform poorly if they assume that individual spatial barcodes contain sufficient UMIs to be clustered into a cell type. Third, submicrometer-resolution technologies inform us of subcellular transcriptomic architecture within individual cells (Cho *et al.*, 2021), but existing tools do not account for subcellular components in their analysis and visualization to accommodate the ultra-high resolution from recent technologies.

To address these challenges, we developed STtools, which is capable of handling various spatial transcriptomics (ST) platforms, including submicrometer-resolution ST platforms such as Seq-Scope. STtools provides a comprehensive framework for analyzing ST datasets, enabling both super-cellular, cellular and sub-cellular resolution analysis and visualization.

## 2 Methods

**Fig. 1.**
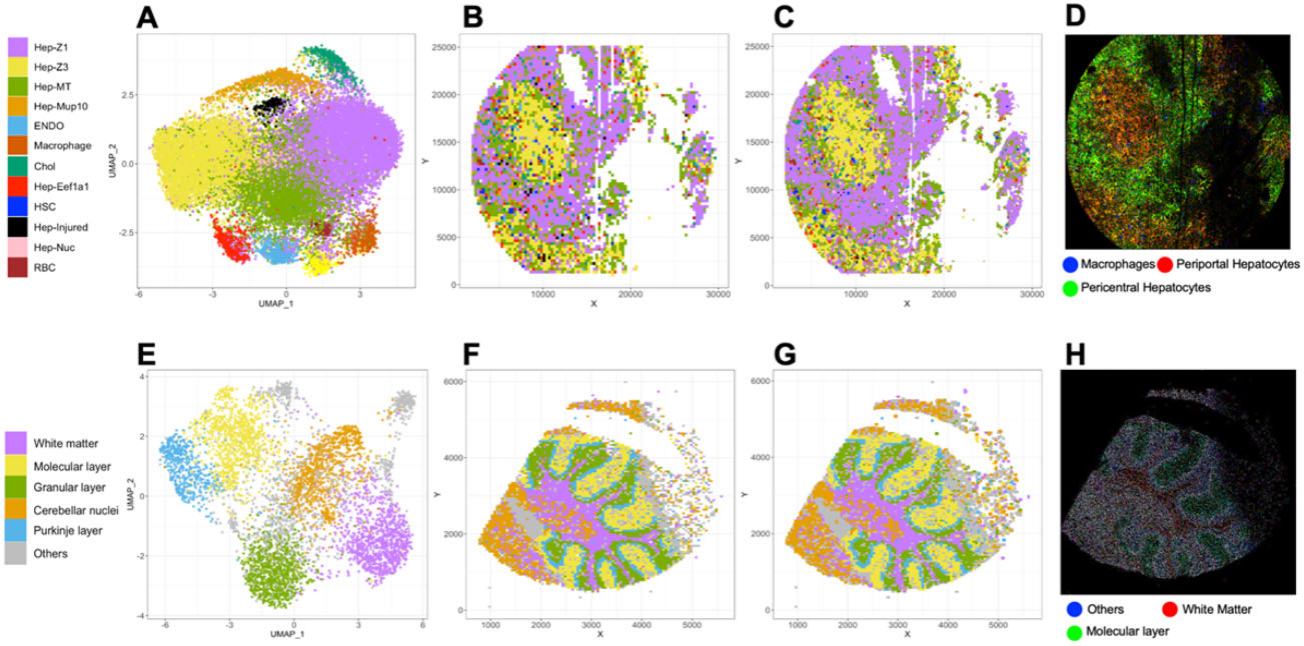
Visualization of ST data with STtools. (**A–D**) Visualize Seq-Scope mouse liver dataset, and (**E–H**) visualize Slide-seq mouse cerebellum dataset. (A, E) Visualize UMAP coordinates and clustered cell types for each squared grid. (B, F) Visualize the clustered cell types for each simple grid (10 μm for Seq-Scope, 50 μm for Slide-seq). (C, G) Visualize the cell types from MSSW with higher resolution (2 μm for Seq-Scope and 10 μm for Slide-seq). (D, H) Visualize selected marker genes in RGB color at ultra-high resolution (1 μm/pixel for Seq-Scope and 10 μm/pixel for Slide-seq)

STtools is able to process ST data from various platforms including, but not limited to, Seq-Scope, Slide-seq and VISIUM. STtools provides a complete solution from raw FASTQ file preprocessing to automated downstream analysis with the flexibility to run the pipeline end-to-end automatically. It also allows users to run a specified set of consecutive steps, or to run individual steps separately. For example, users can skip the FASTQ processing steps and instead start from a spatial gene expression matrix for downstream analysis such as clustering and visualization using STtools. STtools workflow currently performs three major tasks—alignment, clustering and visualization—consisting of eight individual steps (Supplementary Fig. S1). The alignment step performs quality control (QC), alignment and spatial expression matrix generation from raw sequence data. The clustering steps perform cell-type clustering in multiscale resolution. The visualization steps visualize the ST data from multiple different perspectives as illustrated (Supplementary Fig. S1, Fig. 1).

The full STtools workflow starts with taking two sets of raw sequence reads in FASTQ format. The first FASTQ file (1st-seq) contains spatial barcode sequences associated with spatial coordinates that are encoded in their Illumina sequence identifiers (Line 1 of FASTQ reads). The second FASTQ file (2nd-seq) contains cDNA sequences from transcripts, attached with the spatial barcodes (Supplementary Fig. S1). After performing initial QC to inspect the distribution of spatial coordinates of barcodes, using these two sets of FASTQ files, STtools aligns each cDNA sequence to the reference genome using STARsolo (Kaminow *et al.*, 2021). Each aligned 2nd-seq read in the BAM file is annotated with error-corrected spatial barcodes based on 1st-seq. After the alignment, multiple sets of digital expression matrices are generated focusing on exonic reads only (Gene), exonic and intronic reads together (GeneFull), or by distinguishing spliced and unspliced reads (Velocyto).

STtools takes digital expression matrices annotated with spatial coordinates, either from the steps above or from external sources, to aid interpretation of the data through barcode aggregation, clustering and visualization. Aggregation across nearby spatial barcodes is particularly important for submicrometer resolution ST technologies and will help infer cell types accurately. However, it may compromise the subcellular resolution attainable by the technology. To support clustering at cellular/subcellular level while keeping the details of high spatial resolution, STtools employs two different spatial aggregation (i.e. binning) algorithms: simple aggregation and multiscale sliding-window (MSSW) aggregation. The simple aggregation method generates a set of non-overlapping, equal-sized bins to capture enough transcripts to be used for cell-type clustering. MSSW generates a set of overlapping bins for finer resolution cell-type identification and visualization (Supplementary Fig. S2). This two-track approach seamlessly and efficiently integrates with Seurat (Hao *et al.*, 2021), so that simple aggregation is used for clustering cell types and MSSW aggregated bins are used to assign cluster types at a finer resolution (Supplementary Fig. S1D–F). STtools also generates high-resolution (<1 μm^2^/pixel) images where red/green/blue (RGB) colors quantify specific arbitrary marker gene sets to help investigators understand the raw spatial gene expression without sacrificing the resolution. Comparing other tools that can handle high-resolution spatial transcriptomic data, STtools offers more comprehensive coverage across various user cases, particularly for SeqScope data (Table 1).

**Table 1.** Comparison between STTools and other related tools (spacemake and squidpy)

Functionality	Spacemake	Squidpy	STtools
Preprocess 1st-Seq FASTQ to prepare alignment (Step A1)	X	X	O
Quality control of spatial coordinates and tissue boundary detection (Step A2)	X	X	O
Aligns the transcriptomic sequence reads and produces spatial expression matrix in standard format (Step A3)	O	X	O
Grid-based simple spatial segmentation (Step C1)	O	X	O
MSSW segmentation (Step C2)	X	X	O
Clustering of each segment (Step C3)	X	O	O
High-resolution visualization of selected genes (Step V1)	X	X	O
Compatible with SlideSeq	O	O	O
Compatible with SeqScope	△	△	O
Provides an end-end solution (including alignment, clustering and visualization)	X	X	O
Allows running individual steps separately	X	O	O
Quantifies both spliced and unspliced reads for subcellular analysis	X	X	O

## 3 Results

We applied STtools to multiple spatial transcriptomic platforms, including Seq-Scope, Slide-seq and VISIUM. We illustrated example results from Seq-Scope mouse liver dataset (Fig. 1A–D) and Slide-seq mouse cerebellum dataset (Fig. 1E–H), which have ∼0.8 and ∼10 μm distance between adjacent spatial barcodes, respectively. We first applied simple square barcode aggregation (100 μm^2^ for Seq-Scope, 2500 μm^2^ for Slide-seq) and then estimated their cell types and UMAP manifolds (Fig. 1A and E). STtools is featured at MSSW analysis by accumulating reads counts in a smaller square grid, to enhance resolution via sliding grid strategy. Using MSSW, we produced 25-fold finer resolution spatial map (4 μm^2^ for Seq-Scope and 100 μm^2^ for Slide-Seq) and performed high-resolution cell-type identification by high-dimensional projection implemented in Seurat (Fig. 1C). As a result, the spatial cluster map from MSSW algorithm provides finer cell-type boundaries than simple barcode aggregation (Fig. 1B) for Seq-Scope dataset. On the other hand, for SlideSeq, the benefit of MSSW was not visually pronounced primarily due to the low resolution of the technology. (Fig. 1F and G).

We also produced ultra-high-resolution spatial RGB geneset plots that visualize the expressions of selected marker gene sets with STtools to visualize customized spatial maps based on user-defined genes. This RGB plotting tool is capable of separating spliced and unspliced reads, and we were able to visualize both cell-type differences (periportal versus pericentral hepatocytes versus macrophages; Fig. 1D, Supplementary Fig. S3A) as well as subcellular differences (e.g. nucleus versus mitochondria versus macrophages; Supplementary Fig. S3C) at a resolution of 1 μm^2^/pixel. These plots can help investigators interpret ST data at an ultra-high resolution to understand subcellular architecture or infiltration of non-parenchymal cell types.

STtools also generates additional visualization of ST data such as the distribution of UMIs across spatial coordinates (Supplementary Fig. S4A) or violin plots of gene counts, UMI counts or fraction of mitochondrial genes (Supplementary Fig. S4B–D) by seamless connection to other single-cell or ST software such as STARsolo (Kaminow *et al.*, 2021), Seurat (Hao *et al.*, 2021) and seqtk (Li, 2021). The digital expression matrix generated by STtools follows the widely used format from 10× Genomics can be directly read from other software tools such as Seurat (Hao *et al.*, 2021) or squidpy (Palla *et al.*, 2022) (Supplementary Fig. S5). STtools also offers a functionality to run Bayespace (Zhao *et al.*, 2021) for VISIUM data to enhance its resolution.

STtools is designed to efficiently process spatial transcriptomic data scaling with millions of spatially resolved barcodes. The total computational cost to process the SeqScope data consisting of 15M spatial barcodes and 1.9 billion raw sequence reads across all stages was modest, taking ∼16 h in an high performance computing (HPC) cluster with 6 3.0 GHz Intel Xeon Gold 6154 CPUs with 30 GB of RAMs for the mouse liver dataset. The cost was orders of magnitude smaller for lower-resolution datasets such as Slide-seq or VISIUM. To compare the computational efficiency of STtools with spacemake, we ran both tools with the same mouse liver SeqScope data (GSM5212844). Spacemake could not handle the full 2nd-seq data with 625.1G bases, so we ran experiments for a subset (SRR14082757) with 104.7G bases. As shown in Table 1, spacemake approximately corresponds to STtools steps A1 to A3, which produces the spatial gene expression matrix after alignments. Spacemake does not have the functionality to extract spatial coordinates from 1st-seq data of SeqScope, so we used the coordinates generated by STtools step A1. It took 254 min to run STtools steps A1–A3, while spacemake took 1100 min. Both pipelines were executed with –-cores 8 option and ran locally on an HP DL380 server with Dual Intel Xeon-G 5118 processor (24 physical cores). The usage of STARsolo instead of STAR and efficiencies in intermediate file generation resulted in significant runtime differences between two pipelines for high-resolution SeqScope data.

## 4 Discussion

In summary, STtools is a comprehensive software pipeline that allows users to align, cluster and visualize spatial transcriptome sequence data generated at submicrometer resolution. Especially, STtools improves the resolution of spatial inference compared to typical segmentation-based approach by leveraging MSSW algorithm. The spatial expression matrix, spatial segmentation and clustering results produced by STtools can be easily fed to other software tools widely used for downstream analysis, such as Seurat (Hao *et al.*, 2021) and squidpy (Palla *et al.*, 2022).

While STtools offers all-in-one analysis to translate raw sequence reads into spatial expression matrix and clustering, it also provides options to perform step-by-step analyses so that the investigators can perform sanity check from each step and adjust the parameters as needed. Users can customize many parameters during the alignment and clustering, including adapter sequences to trim, reference genomes to align and the thresholds to filter genes and spatial segments before clustering. Users can always load the spatial expression matrix generated by STtools in a standard format to perform more tailored analysis on their own using Seurat, squidpy or other downstream software tools.

Although higher-resolution spatial inference can be made by the MSSW algorithm, compared to other standard spatial transcriptomic analysis tools, it still has rooms for improvement. Due to the limited number of UMIs per region, each spatial segment still needs to be larger than subcellular compartments (e.g. ∼10 µm), so subcellular analysis with MSSW is not feasible. Spatial smoothing algorithms that deliver robust inference for extremely sparse expression profiles per spatial unit will be needed to enable truly subcellular inference beyond visualization of subcellular compartments.

There are many more improvements that can be made to STtools in the future. For example, methods to impute spatial expression profiles (Shengquan *et al.*, 2021), methods to jointly cluster cellular and subcellular components together, or methods to automatically overlay histological images and spatial expressions are useful features that can be added in the next major updates of STtools.

## Supplementary Material

vbac061_Supplementary_Data

## Data Availability

The data used to generate images in this paper is available at the Gene Expression Omnibus (GEO) database with accession number GSE169706.
