# Supplementary material for: STtools: a comprehensive software pipeline for ultra-high-resolution spatial transcriptomics data
Source: Bioinform Adv. 2022 Sep 1;2(1):vbac061. doi: 10.1093/bioadv/vbac061 (PMC9590442; doi:10.1093/bioadv/vbac061)

Figure S1. STtools Workflow

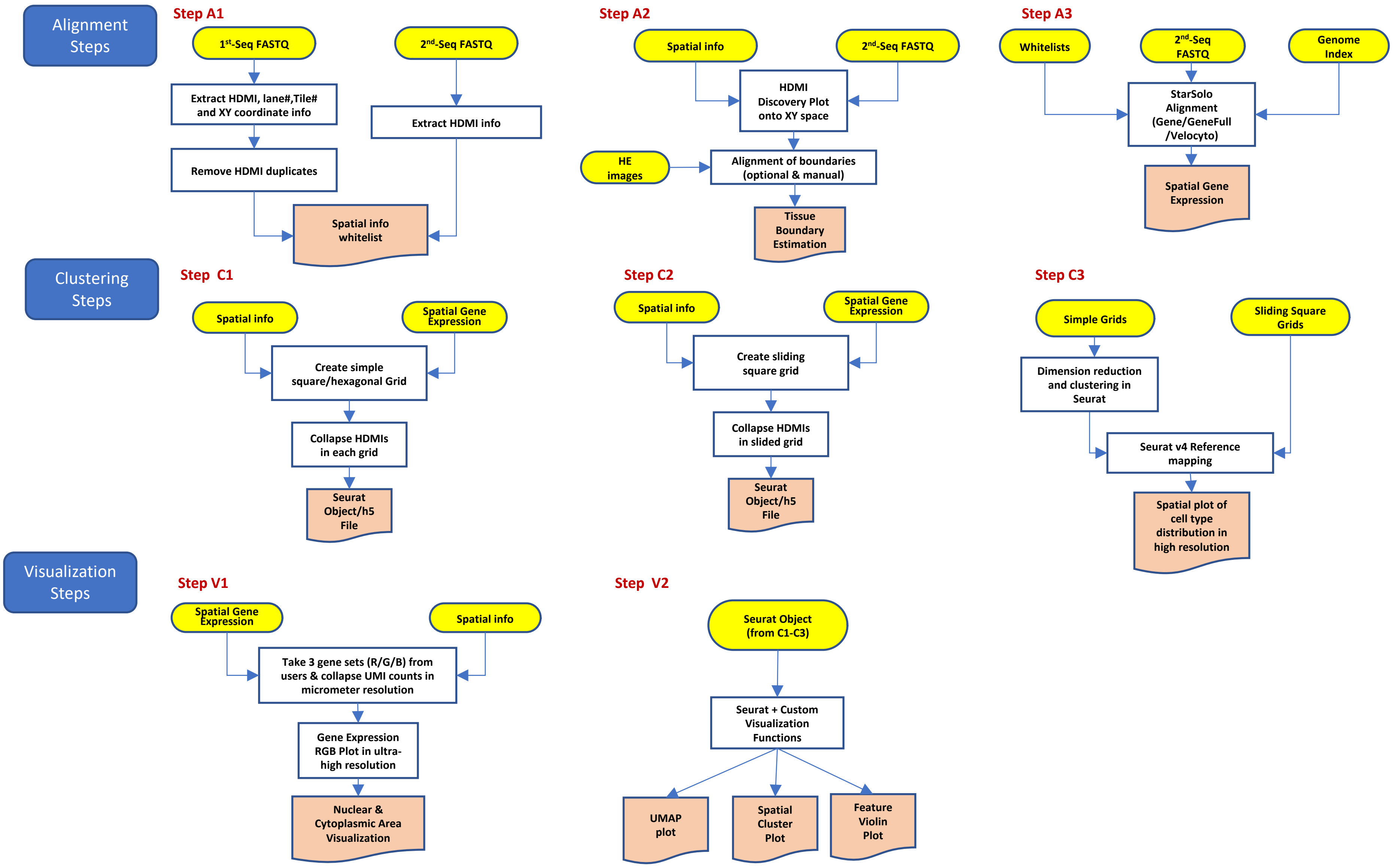

*HDMI: High-Definition Map coordinate Identifier (Cho *et al*. 2021)

STtools workflow consists of three major steps – (A) Alignment steps, (C) Clustering steps, and (V) Visualization steps. The alignment steps starts with two sets of raw FASTQ files to extract spatial coordinates to be used for alignment (A1), visualizes the distribution of spatial barcodes (A2), and perform alignment with STARsolo (A3). The clustering step starts with simple square grid approach to perform initial clustering (C1), followed by multi-scale sliding window (MSSW) to generate high-resolution grids (C2), whose cell types are projected from the initial clusters onto high-resolution grids with Seurat (C3). The visualization steps generates ultra-high-resolution RGB plots for user-specified marker gene sets (V1). It also produces UMAP plots, Cluster plots along spatial coordinates, and feature plots for basic QC metrics or user-specified genes by leveraging Seurat (V2)

Figure S2. Multi-scale sliding window (MSSW) Algorithm

Algorithm: Multi-scale Sliding Window (MSSW) algorithm

```
Data: D[x, y, z]: gene expression counts of pixel at (x, y) for gene z,
      1 ≤ x ≤ w, 1 ≤ y ≤ h, 1 ≤ z ≤ g.
m_x, m_y : the width and height of a grid
s_x, s_y : the unit of sliding window step.
Result: (w/s_x) × (h/s_y) image I with each pixel having a cluster id
for y ← m_y/2 to h − m_y/2 by s_y do
    for x ← m_x/2 to w − m_x/2 by s_x do
        d ← {0}^g;
        for k_y ← y − m_y/2 + 1 to y + m_y/2 do
            for k_x ← x − m_x/2 + 1 to x + m_x/2 do
                d ← d + D[k_x, k_y, :];
            end
        end
        I[x/s_x, y/s_y] ← refmap(d);
    end
end
/* refmap():N^g → N, maps a Seurat object (with g genes) to
   one of reference clusters identified by simple grids.
   This function can be easily replaced with other mapping
   functions.                                                */
/* All unmapped pixels at the outside boundary region will
   be padded with the nearest mapped                         */
```

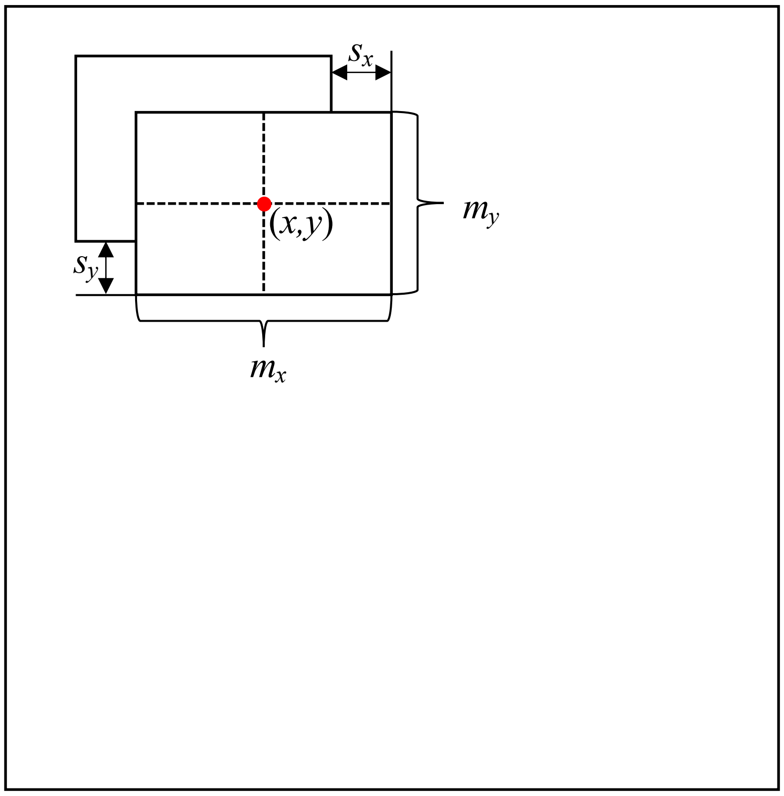

Figure S3. Spatial RGB visualization of marker gene sets by STtools

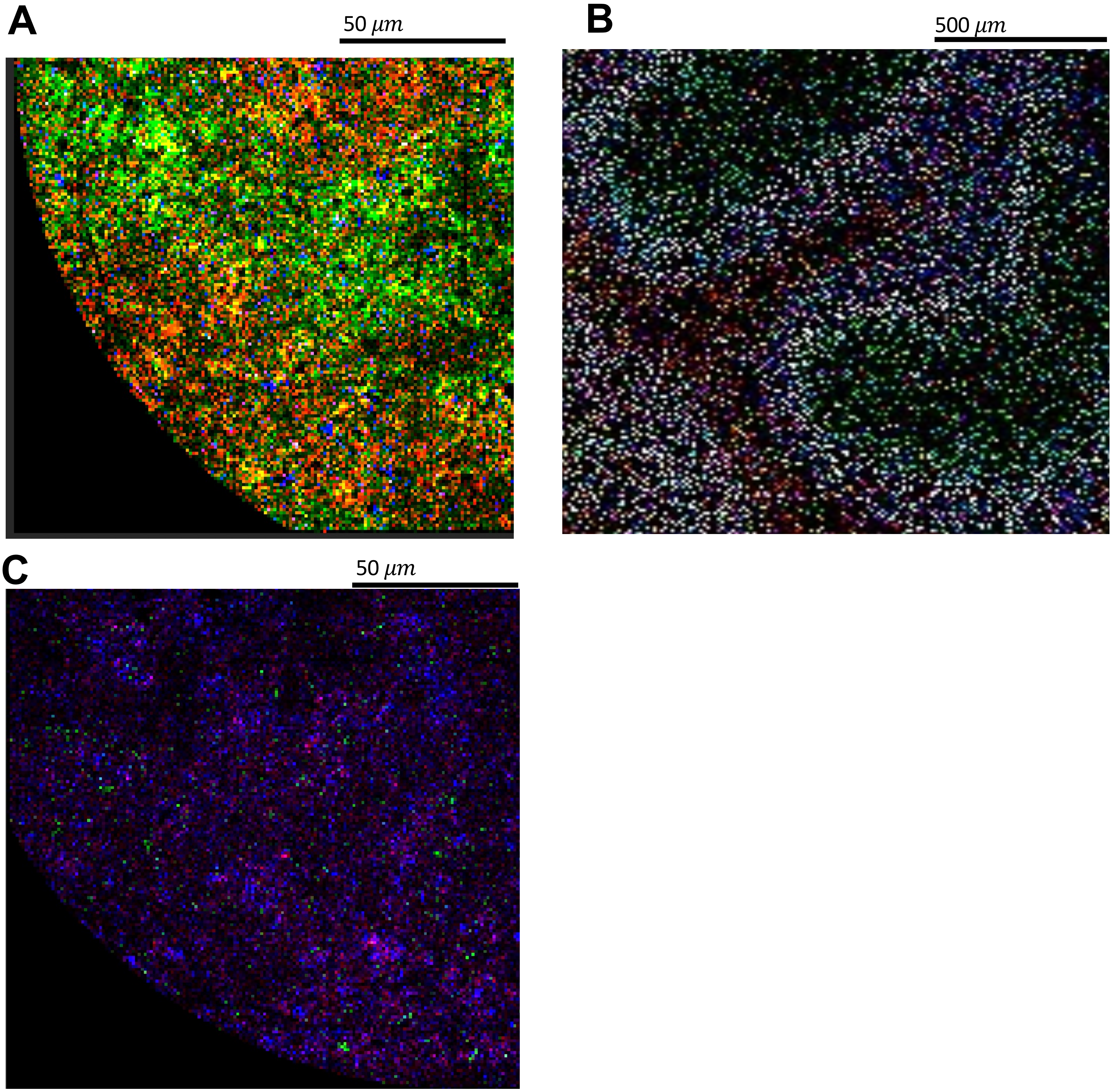

Spatial RGB visualization of marker gene sets by STtools. Zoomed-in from Figure 1D and 1H. (A) magnifies the spatial map of Seq-Scope mouse liver from Figure 1D, visualizing unspliced reads(blue), periportal hepatocyte(red), and pericentral hepatocyte(green). (B) magnifies the spatial map of Slide-seq mouse cerebellum from Figure 1H, visualizing white matter (red), molecular layer (green) and other cell types (blue). (C) magnifies the spatial map of Seq-Scope mouse liver from Figure 1D (same region to A), visualizing mitochondrial RNAs (red), macrophages (green – same to blue in A), unspliced reads (blue).

Figure S4. Additional visualization of spatial transcriptomics data produced by STtools

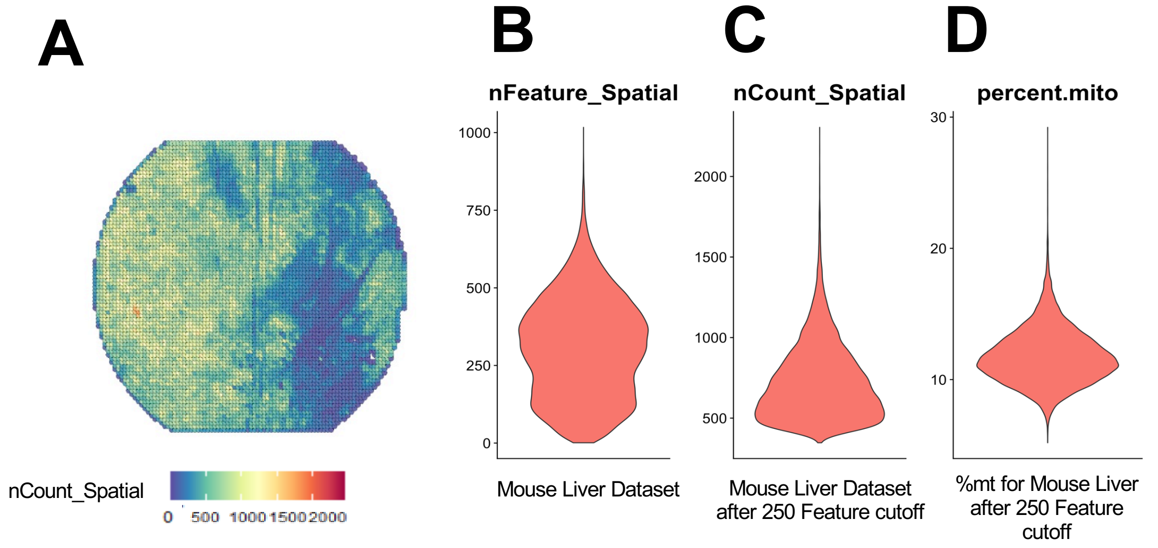

Additional visualization of spatial transcriptomics data produced by Sttools. (A) –(D) are generated with Seq-Scope mouse liver data(ref). (A) visualizes the spatial distribution of total UMIs per simple square grid (10 μm). (B)-(D) visualizes the the distribution of (B) gene counts (C) total UMIs, and (D) percentage of mitochondrial genes per each grid. (C) and (D) only visualizes grids with more than 250 genes expressed.

Figure S5. Connecting STtools to squidpy with SeqScope liver dataset

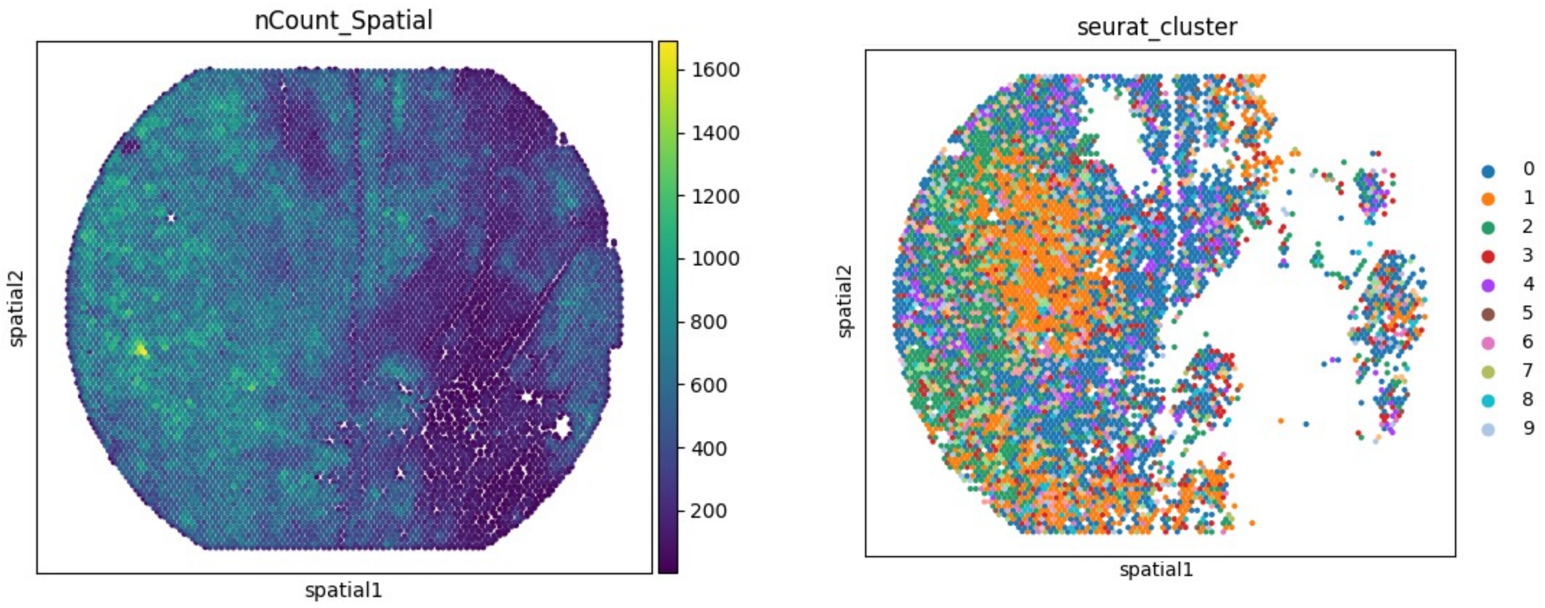

Supplement: vbac061_Supplementary_Data [file vbac061_supplementary_data.pdf]
